# The RNA Methylation Modification 5-Methylcytosine Impacts Immunity Characteristics, Prognosis and Progression of Oral Squamous Cell Carcinoma by Bioinformatics Analysis

**DOI:** 10.3389/fbioe.2021.760724

**Published:** 2021-12-09

**Authors:** Li Gao, Ru Chen, Masahiro Sugimoto, Masanobu Mizuta, Lei Zhou, Yo Kishimoto, Xinsheng Huang, Koichi Omori

**Affiliations:** ^1^ Department of Otolaryngology, Head and Neck Surgery, Graduate School of Medicine, Kyoto University, Kyoto, Japan; ^2^ Department of Medicine, Matsusaka City Hospital, Matsusaka, Japan; ^3^ Research and Development Center for Minimally Invasive Therapies Health Promotion and Preemptive Medicine, Tokyo Medical University, Tokyo, Japan; ^4^ Department of Otolaryngology-Head and Neck Surgery, Shanghai Zhongshan Hospital Affiliated to Fudan University, Shanghai, China

**Keywords:** 5-methylcytosine (m5C), RNA methylation modification, oral squamous cell carcinoma, tumor immune microenvironment, prognosis

## Abstract

Disorders pertaining to 5-methylcytosine (m5C) modifications are involved in the pathological process of many diseases. However, the effect of m5C on the tumorigenesis and progression of oral squamous cell carcinoma (OSCC) remains unclear. In this study, we integrated the genomic and clinical data of 558 OSCC samples to comprehensively evaluate m5C modification patterns. Based on 16 m5C methylation regulators, two m5C modification clusters were identified with distinct tumor immune microenvironment (TIME) characteristics and prognosis in OSCC. We then performed weighted gene co-expression network analysis (WGCNA) to identify m5C modification cluster-related modules. Genes in the selected module were chosen to construct the m5Cscore scoring system for evaluating m5C modification pattern in individual OSCC patients. Patients with a high m5Cscore had higher immune, stromal, and ESTIMATE scores; lower tumor purity score; lower immune activity; and higher tumor mutational burden. The overall survival rate and progression-free survival rate were markedly worse and the tumor recurrence rate was higher in OSCC patients with a high m5Cscore. Furthermore, patients with oral leukoplakia who also had a high m5Cscore had a higher risk of deterioration to OSCC. This study demonstrated that m5C modification patterns might affect the TIME in OSCC. m5Cscore may provide a new approach for predicting the prognosis and progression of OSCC.

## Introduction

5-methylcytosine (m5C) is one of the longest-known post-transcriptional RNA methylation modifications and widely exists in mRNA, rRNA, and tRNA transcripts (Van Haute et al., 2019) (Schumann et al., 2020)(Bohnsack et al., 2019). m5C plays a crucial regulatory role in various cellular processes, including RNA processing, protein translational regulation, and stress responses (Liu et al., 2017). At the post-transcriptional level, m5C modification is dynamically regulated by mediator proteins known as “writers,” “erasers,” and “readers” (Trixl and Lusser, 2019). The “writers” comprise methyltransferase including NOP2, NSUN2-7, DNMT1, DNMT3A, DNMT3B, and TRDMT1, which add a methyl group at the C5 position of RNAs. The “erasers” are TET2, TET3 and ALKBH1, which serve as demethylases to remove methyl groups from m5C. The “readers,” such as ALYREF and YBX1, recognize and bind to m5C sites on mRNA (Chen Y. S. et al., 2021) (Sibbritt et al., 2013) (Fu et al., 2014). m5C modification has been verified to involve in the development of many diseases, such as cancers. Chen et al. found that NSUN2 and YBX1 promoted oncogenesis in human bladder urothelial carcinoma by targeting the m5C methylation site in the untranslated region of HDGF3 (Chen et al., 2019a). NSUN2 is reported to upregulate in the human breast carcinoma and colon carcinoma tissues, compared with healthy tissues (Frye and Watt, 2006).

Oral squamous cell carcinoma (OSCC) is the most common epithelial malignancy in the oral area and is characterized by high recurrence rates, poor survival outcomes, and high cervical lymph-node metastasis rates (Gődény, 2014) (Bray et al., 2018) (Warnakulasuriya, 2009). Recent epidemiological investigations indicated that smoking, alcohol consumption, betel-nut chewing, diet, and nutrition are high-risk factors related to the oncogenesis and development of OSCC (Mashberg et al., 1993) (Chen et al., 2008). It is commonly thought that OSCC arises *de novo* or precedes latent lesions in the oral cavity (Choi and Myers, 2008). Leukoplakia is the most common potentially precancerosis in the oral area (Quan et al., 2020). Approximately 0–36% cases of leukoplakia may progress to OSCC (van der Waal, 2014) (Lee et al., 2000) (Chaturvedi et al., 2020). Over the past decades, great achievements have been made in the diagnosis and treatment of OSCC through methods such as imaging technologies, surgery, chemotherapy, molecular targeted therapies, and immunotherapy (Chai et al., 2020) (Kaidar-Person et al., 2018). However, the prognosis of OSCC remains poor, the main reasons for which are metastasis and diagnosis at advanced stages (Zhang et al., 2019). Therefore, further investigation into the mechanisms involved in the oncogenesis and development of OSCC might help improve diagnostic accuracy at an early stage, as well as the treatment effective rate.

It has been verified that dysfunction of the tumor immune microenvironment (TIME) affects the oncogenesis and development of OSCC (Quan et al., 2020) (Bhat et al., 2021). There is growing evidence that OSCC/head and neck squamous cell carcinoma (HNCSS) is highly immunosuppressive, mediated by recruitment of host immunosuppressive cells and inhibitory mediators (Jewett et al., 2006) (Tong et al., 2012). Patients with OSCC usually have worse antigen-presenting ability, lower infiltrating-lymphocyte viability, and lower immune infiltrating cell counts than healthy samples (Lasisi et al., 2013) (Barth et al., 2004) (Grimm et al., 2016). Furthermore, the rapid deterioration of oral leukoplakia into OSCC has also be proven in an immunosuppressed liver transplant recipient (Hernández et al., 2003). Nevertheless, further investigations in the underlying regulatory mechanisms are still needed.

In this study, we integrated the genomic and clinical data of 558 OSCC samples to comprehensively evaluate m5C modification patterns, and we correlated m5C modification patterns with prognosis and TIME characteristics. Weighted gene co-expression network analysis (WGCNA) was performed to identify m5C modification cluster-related modules. Genes in the selected module were chosen to construct the m5Cscore scoring system for evaluating m5C modification pattern in individual OSCC patients. The TIME characteristics and prognosis between m5C modification patterns and m5Cscore groups (high or low) were distinct. The m5C modification patterns and the m5Cscore scoring system might help in the prediction of the prognosis and progression of OSCC.

## Materials and Methods

### Data Processing

The mRNA expression dataset and clinicopathological characteristic of OSCC patients were extracted from The Cancer Genome Atlas (TCGA) (https://portal.gdc.cancer.gov/) and the NCBI GEO database (https://www.ncbi.nlm.nih.gov/geo/). In total, 558 OSCC samples, 32 normal samples, and 101 oral leukoplakia samples were screened out for further evaluation. 322 OSCC samples and 32 normal control samples were extracted from TCGA-HNSCC cohort, the cancerous sites included alveolar ridge, hard palate, floor of the mouth, the base of the tongue, oral cavity, buccal mucosa, and oral tongue. GSE65858 includes 83 OSCC patients; GSE41613 contains 97 OSCC samples; GSE26549 includes 86 oral leukoplakia patients; GSE31056 includes 22 OSCC patients; and GSE85195 contains 34 OSCC patients and 15 oral leukoplakia patients. Detailed information on these datasets is provided in Supplementary Table S1. The clinicopathological information of OSCC patients is provided in Supplementary Table S2. The data processing were performed as we previously described (Gao et al., 2021). To remove the batch effects, the RNA sequencing data (FPKM format) data of OSCC patients in TCGA HNSCC was transformed into transcripts per kilobase million (TPM) format and then combined with microarray data in GSE65858. Copy number variations (CNVs) were obtained from the UCSC Public Hub and analyzed using the RCircos package in R. Somatic mutation expression data were downloaded from TCGA and analyzed using the maftools package in R.

### Unsupervised Consensus Clustering Analysis

We conducted consensus clustering analysis to classify OSCC patients into distinct m5C-modification clusters using the ConsensusClusterPlus R package. In total, 16 m5C methylation regulators, including “writers” (DNMT1, DNMT3A, DNMT3B, NOP2, NSUN2-7, and TRDMT1), “erasers” (ALYREF and YBX1), and “readers” (TET2, TET3 and ALKBH1) retrieved from literature were analyzed in this study (Chen et al., 2021c) (Trixl and Lusser, 2019) (Bohnsack et al., 2019). Clustering heatmaps were plotted using the pheatmap package.

### Estimation of Tumor Immune Microenvironment Characteristics

We conducted single-sample gene set enrichment analysis (ssGSEA) to quantify the relative amount of 27 immune cell types in the TIME of OSCC using the GSVA and GSEABase packages. These gene sets acquired from recent studies were provided in Supplementary Table S3 (Charoentong et al., 2017) (He et al., 2018). ESTIMATE method was used to estimate the proportions of stromal cells (SCs), immune cells (ICs) in the TIME of OSCC. Tumor purity was predicted based on the SCs, ICs, and ESTIMATE scores. The deconvolution approach CIBERSORT was used to investigate the differences in 22 immune cell subtypes among the m5C-modification clusters. The “signature matrix” of 547 genes was acquired from CIBERSORT (https://cibersort.stanford.edu/index.php), namely, LM22.txt. The differences in physiological processes in different m5C-modification clusters were evaluated by gene set variation analysis (GSVA). Gene sets “c2.cp.kegg.v7.4.symbols” acquired from the MSigDB (http://www.gsea-msigdb.org/gsea/msigdb) were used for the GSVA analysis.

### Construction of Gene Co-expression Network

We used WGCNA to explore m5C-modification cluster-related modules. Based on the top 50% of the most variable genes (7,949 genes), the gene co-expression network was constructed. The correlation of the expression of these filtered genes was calculated, and *β* = 6 was selected as optimal soft threshold for building a scale-free topological network. Topological overlap matrix (TOM) analysis was performed to cluster the adjacency matrix of gene expression. The modules were identified with the criterion set as the mini-size of module gene numbers was 40, and a cut height of 0.9. The module with the strongest relationship with m5C-modification clusters was selected for further analysis.

### Generation of m5Cscore

M5Cscore was adopted for the quantitative analysis of m5C-modification patterns in an individual patient with OSCC. This method was established as we previously described (Gao et al., 2021). Briefly, the prognostic value of each gene in the selected module was evaluated by Cox regression analysis. Subsequently, principal component analysis (PCA) was conducted to calculate the principal components (PCs) 1 and 2, which were used for m5Cscore calculations.

$$\mathrm{m5Cscore}=\sum \left(\mathrm{PC}1_{i}+PC2_{i}\right)$$ (1)

where _i_ is the expression of the selected genes.

### Statistical Analysis

All statistical analyses were implemented using R software version3.5.3 (https://www.r-project.org/). The Kaplan–Meier analysis and log-rank analysis were performed to evaluate the progression-free survival (PFS) and overall survival (OS) between clusters. We conducted Pearman correlation analysis to explore the correlation between m5Cscore and known gene signatures and the correlation coefficient of gene expression. Biological gene signatures were retrieved from previous studies (Couzin-Frankel, 2013) (Melero et al., 2021). Student’s *t*-test was used to evaluate the statistical differences between two groups. Kruskal-Wallis tests and one-way ANOVA tests were performed when there were more than two groups. In all cases, the *p*-value was two-sided, and a *p*-value < of 0.05, was considered statistically significant.

## Results

### Expression and Prognostic Value of m5C Methylation Regulators in OSCC

**FIGURE 1:**
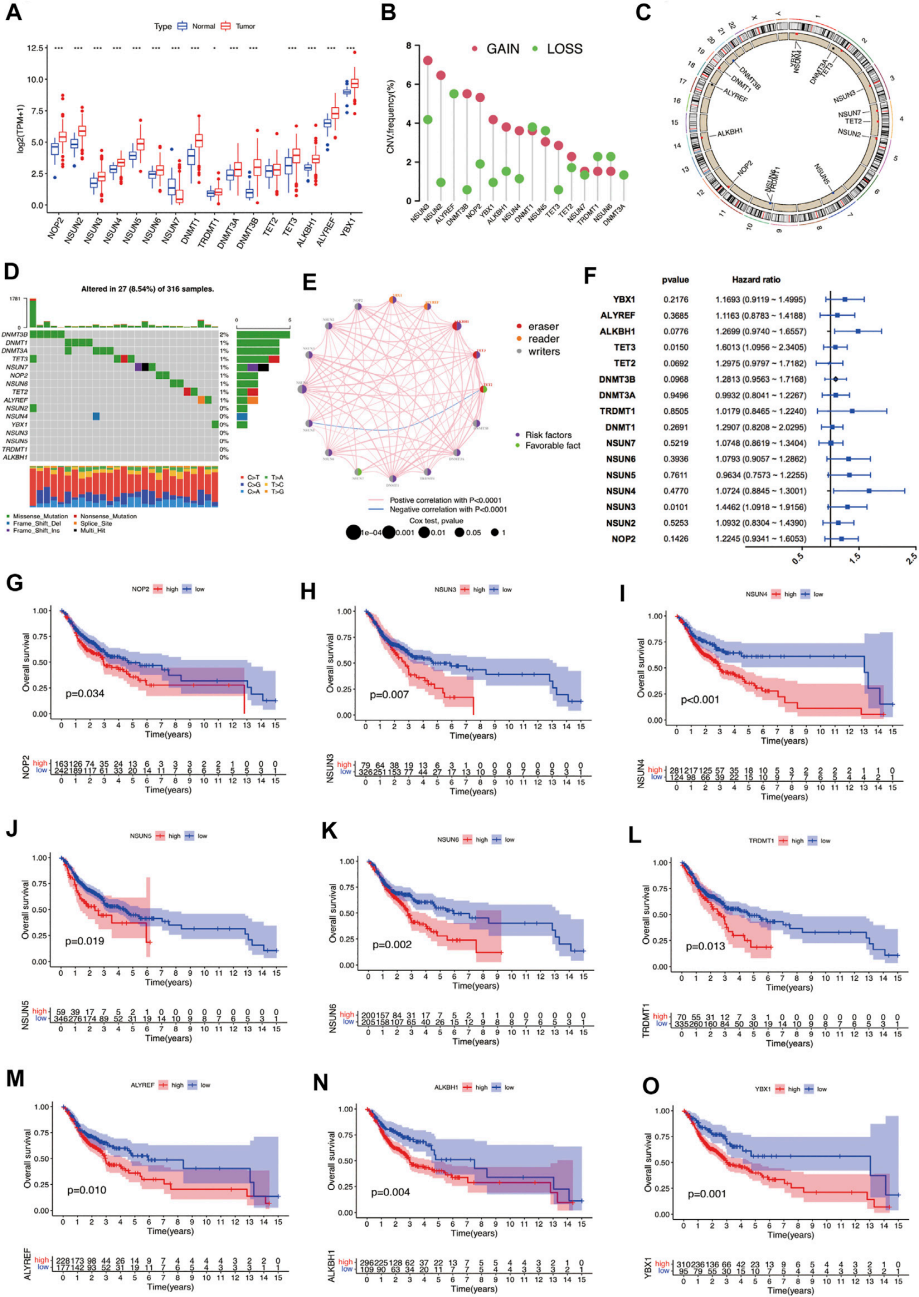
Overview of genetic and prognostic information of m5C regulators in OSCC. **(A)** Expression of 16 m5C regulators between OSCC and normal samples. (*p < 0.05; ***p < 0.001). **(B)** Frequency plots of copy number alteration defined in all m5C regulators. **(C)** Location of copy number variations of m5C regulators on chromosomes. **(D)** Waterfall plot of the mutation genes and types of m5C regulators in OSCC. **(E)** Interaction between m5C regulators in OSCC. **(F)** Prognostic analyses of m5C regulators by univariate Cox regression test. **(G–O)** OS analysis of m5C regulators in OSCC patients. m5C, 5-methylcytosine; OSCC, oral squamous cell carcinoma.

In this study, sixteen m5C RNA methylation regulators, including “writers” (DNMT1, DNMT3A, DNMT3B, NOP2, NSUN2-7, and TRDMT1), “erasers” (ALYREF and YBX1), and “readers” (TET2, TET3 and ALKBH1) were analyzed to analyze the expression and prognosis significance of m5C RNA methylation regulators in OSCC. Between OSCC samples and normal controls, the expression levels of m5C regulators were significantly different, except for TET2 (Figure 1A). The expression of NSUN7 was markedly downregulated in OSCC samples, while that of other regulators was significantly upregulated. Among these m5C regulators, CNV alterations were relatively prevalent. NSUN3, NSUN2, DNMT3B, NOP2, YBX1, ALKBH1, NSUN4, and TET3 were predominantly amplified, while ALYREF had a higher frequency of CNV deletion (Figures 1B,C). The frequency of somatic mutations in DNMT3B, DNMT1, DNMT3A, TET3, NSUN7, NOP2, NSUN6, TET2, and ALYREF was >1%. However, among the 316 OSCC samples, only 27 samples (8.54%) showed m5C regulators mutation (Figure 1D). The aforementioned findings suggest that there is a marked difference between OSCC and normal controls in the expression and genetic variations in m5C regulators.

We also evaluated the prognostic value of m5C regulators in OSCC (Figures 1E,F). Most m5C regulators showed tumor-promoting effects, except for TET2 and NSUN7. The high expression of nine m5C regulators suggested poorer survival outcomes in OSCC patients: NOP2 (*p* = 0.034), NSUN3 (*p* = 0.007), NSUN4 (*p* < 0.001), NSUN5 (*p* = 0.019), NSUN6 (*p* = 0.002), TRDMT1 (*p* = 0.013), ALYREF (*p* = 0.010), ALKBH1 (*p* = 0.004) and YBX1 (*p* = 0.010) (Figures 1G–O). In the validation cohort GSE41613, the high expression of seven m5C regulators suggested worse outcomes, namely NSUN2 (*p* = 0.010), NSUN3 (*p* = 0.046), TRDMT1 (*p* = 0.032), DNMT3A (*p* = 0.046), DNMT3B (*p* < 0.001), ALKBH1 (*p* < 0.001), and ALYREF (*p* < 0.001); however, high expression of TET2 (*p* = 0.011) was related to better prognosis (Supplementary Figure S1). These findings indicate that crosstalk among m5C regulators might affect the formation and effect of distinct m5C modification clusters and might further affect the oncogenesis, development, and prognosis of OSCC.

### Depiction of m5C-Modification Clusters and Related Biological Functions in OSCC

**FIGURE 2:**
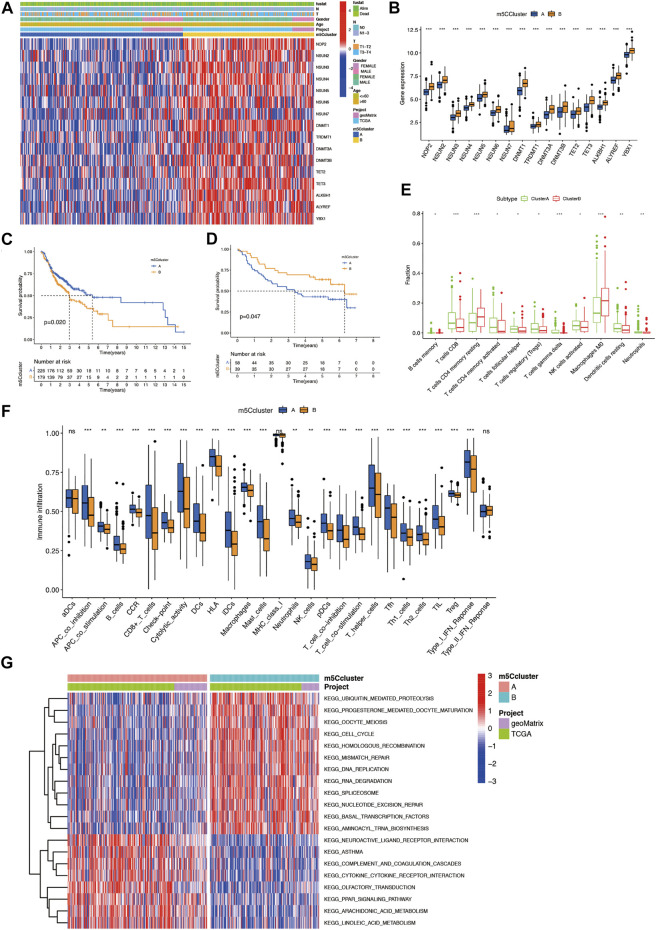
Construction of two m5C modification clusters in OSCC. **(A)** Expression of 16 m5C regulators and related clinical information in OSCC. **(B)** Comparison of gene expression of m5C regulators between two clusters. **(C)** OS analysis for OSCC patients within different clusters. **(D)** OS analysis for OSCC patients within different m5C modification clusters in the validation cohort. **(E)** CIBERSORT analysis of the expression of lymphocytes between two m5C modification clusters. **(F)** The abundance of immune infiltrating cells between two m5C modification clusters by ssGSEA analysis. **(G)** GSVA enrichment analysis of the biological activities between two m5C modification clusters. *p < 0.05; **p < 0.01; ***p < 0.001; ns = not significant. m5C, 5-methylcytosine; OS, overall survival; OSCC, oral squamous cell carcinoma.

To comprehensively evaluate the association among these m5C regulators, we conducted consensus clustering analysis to stratify 405 OSCC patients into two independent clusters: clusters A and B (Figure 2A; Supplementary Figures S2 A–C). Figure 2B shows that the expression of m5C regulators in the two clusters was significantly distinct. The expression of m5C regulators was noticeably higher in cluster B. The OS analysis indicated that compared with cluster A, prognosis of patients in cluster B was markedly poorer (*p* = 0.020) (Figure 2C). What’s more, in GSE41613, OSCC patients were also classified into two clusters (Supplementary Figures S2D–F). Most m5C regulators were highly expressed in cluster A (Supplementary Figure S2G). The OS outcomes in cluster A were worse than those in cluster B (*p* = 0.047) (Figure 2D).

We further evaluated the effect of m5C modification patterns on the TIME in OSCC. CIBERSORT analysis results indicated that compared with cluster A, cluster B had markedly lower fractions of resting dendritic cells (DCs), activated natural killer (NK) cells, neutrophils, and CD8^+^ T cells, and markedly higher fractions of resting memory CD4^+^ T cells and M0 macrophages (Figure 2E). Furthermore, ssGSEA analysis showed compared with cluster A, cluster B had lower immune activity in most immune cell types, included CD8^+^ T cells, tumor-infiltrating lymphocytes (TILs), and B cells, which indicated a deeper immunosuppressed state in cluster B (Figure 2F). GSVA enrichment analysis revealed that cluster B was significantly enriched in ubiquitin-mediated proteolysis, mismatch repair-related pathways, progesterone-mediated oocyte maturation, and cell-cycle. Cluster A was markedly enriched in cytokine-cytokine receptor interaction, neuroactive ligand receptor interaction, and PPAR signaling pathway-related pathways (Figure 2G). These findings imply that m5C-related modification patterns might participate in TIME regulation in OSCC.

### m5C Phenotype-Related Genes in OSCC

**FIGURE 3:**
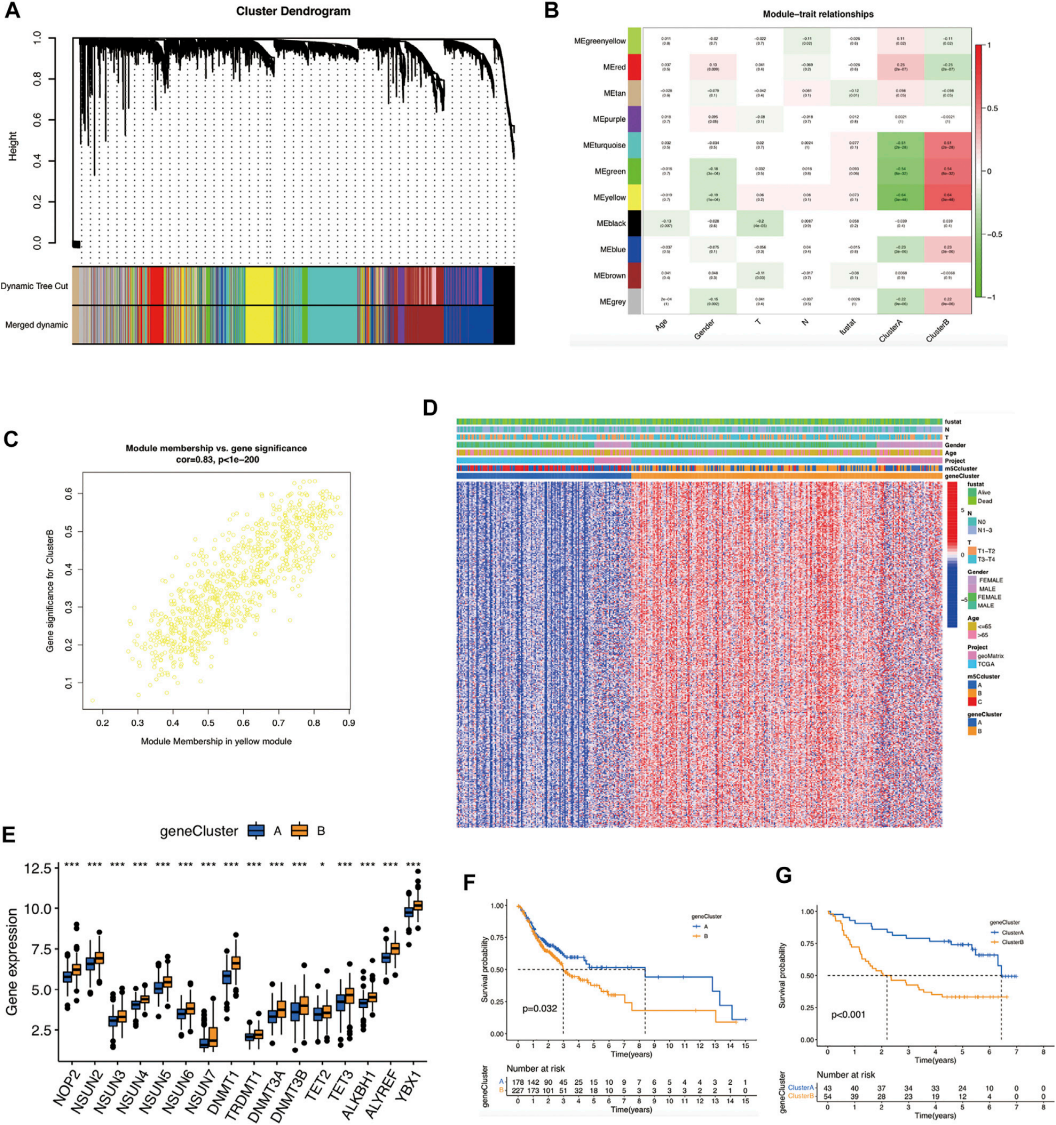
Identification of m5C-modification cluster-related modules and m5Cscore scoring-system construction. **(A)** Construction of hierarchical clustering dendrogram. **(B)** Correlation analysis between modules; the yellow module is most related to the m5C modification cluster. **(C)** A scatter plot of gene significance versus module membership in the yellow module. **(D)** Heat map of the expression of m5C phenotype-related genes and related clinical information in OSCC. **(E)** Comparison of gene expression of m5C regulators between the two m5C gene clusters. (*p < 0.05; ***p < 0.001). **(F)** OS analysis for OSCC patients within different m5C gene clusters. **(G)** OS analysis for OSCC patients within different m5C gene clusters in the validation cohort. m5C, 5-methylcytosine; OS, overall survival; OSCC, oral squamous cell carcinoma.

To identify the m5C-modification cluster-related modules, we constructed a gene co-expression network (Figure 3A). *β* = 6 was selected as the optimal soft threshold for building a scale-free topological network (Supplementary Figure S3A). As illustrated in Figure 3B; 11 modules were identified. The yellow module demonstrated the highest correlation with the m5C cluster (*r* = 0.64, p = 3e-48). In addition, genes in the yellow module were markedly co-expressed (weighted correlation = 0.83, P < 1e-200) (Figure 3C). 53 genes in this module were screened out for further analysis, with the screening criteria of |MM| ≥ 0.8 and |GS| ≥ 0.2 (Supplementary Table S4).

Next, we conducted a consensus clustering analysis based on m5C cluster-related genes. OSCC patients were divided into two m5C gene clusters: gene clusters A and B (Figure 3D; Supplementary Figures S4B–D). Cluster B had the highest expression of most m5C regulators and poorer prognosis (*p* = 0.032) (Figures 3E,F). We verified and obtained the same outcomes in the validation cohort (*p* < 0.001) (Figure 3G; Supplementary Figures S3E–H).

### m5Cscore Scoring System and Related Prognostic and Immune Characteristics in OSCC

**FIGURE 4:**
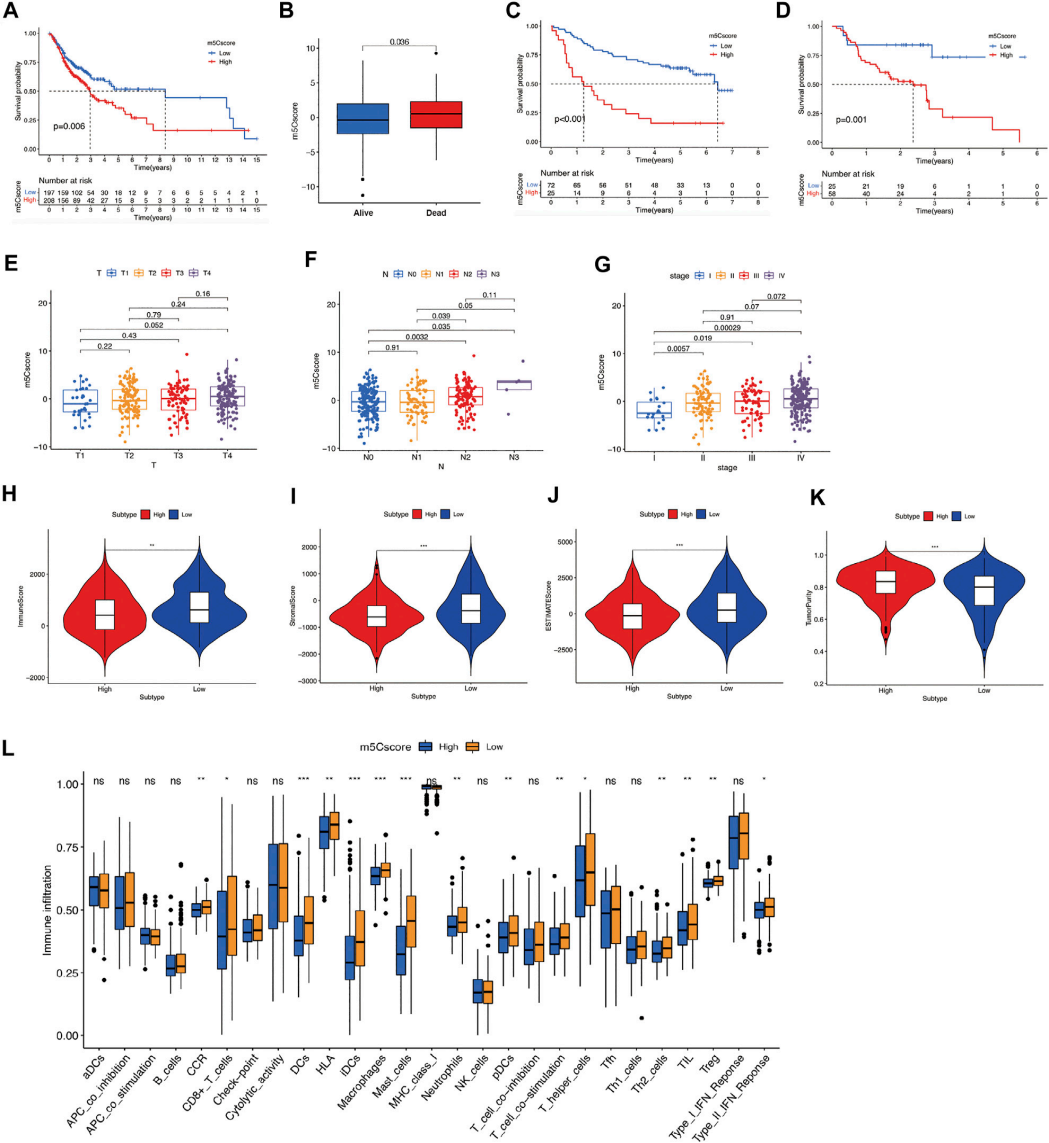
Construction of m5Cscore scoring system. **(A)** OS analysis for OSCC patients in high- or low-m5Cscore groups. **(B)** Comparison of m5Cscore in OSCC patients with different prognoses. **(C)** OS analysis for OSCC patients in high- or low-m5Cscore groups in the validation cohort GSE41613. **(D)** PFS analysis for OSCC patients in high- or low-m5Cscore groups in GSE65858**. (E-G)** Comparison of m5Cscore in OSCC patients at different T **(E)**, N **(F)**, and clinical stages **(G)**. **(H–K)** ESTIMATE analysis for OSCC patients in high- or low-m5Cscore groups, including immune score **(H)**, stromal score **(I)**, ESTIMATE score **(J)**, tumor purity scores **(K)**. **(L)** The abundance of immune infiltrating cells between high- and low-m5Cscore group by ssGSEA analysis. *p < 0.05; **p < 0.01; ***p < 0.001; *ns* = not significant. m5C, 5-methylcytosine; OS, overall survival; OSCC, oral squamous cell carcinoma; PFS, progression-free survival.

We calculated the m5Cscore for individual OSCC patients using PCA. Patients with high-m5Cscore showed poorer OS than those with low-m5Cscore (Figures 4A,B). A consistent prognosis result was verified using GSE41613 (Figure 4C). The PFS of patients in high-m5cscore group was also poorer than those in low-m5Cscore group in GSE65858 (Figure 4D). We further evaluated the m5Cscore between patients in different tumor stages, nodal stages, and clinical stages. As shown in Figures 4E–G, m5Cscore progressively increased with the progression of the T stage (Figure 4E), N stage (Figure 4F), and clinical stage (Figure 4G). At different T and N stages, OSCC patients with a high m5Cscore showed poorer OS (Supplementary Figures S4 A–D). m5Cscore and m5C clusters and m5C gene clusters had a positive correlation, i.e., m5Cscore were higher in m5C cluster B and m5C gene cluster B (Supplementary Figures S4 E–F). In addition, compared with low-m5Cscore group, the SCs, ICs, and ESTIMATE scores in the high-m5Cscore group were noticeably lower, while the tumor purity score was noticeably higher (Figures 4H–K). What’s more, the immune activity of most types of immune cells in the high-m5Cscore group were significantly lower, compared with low-m5Cscore group (Figure 4L), which further suggests an association between a suppressive TIME and high m5Cscore in OSCC patients.

**FIGURE 5:**
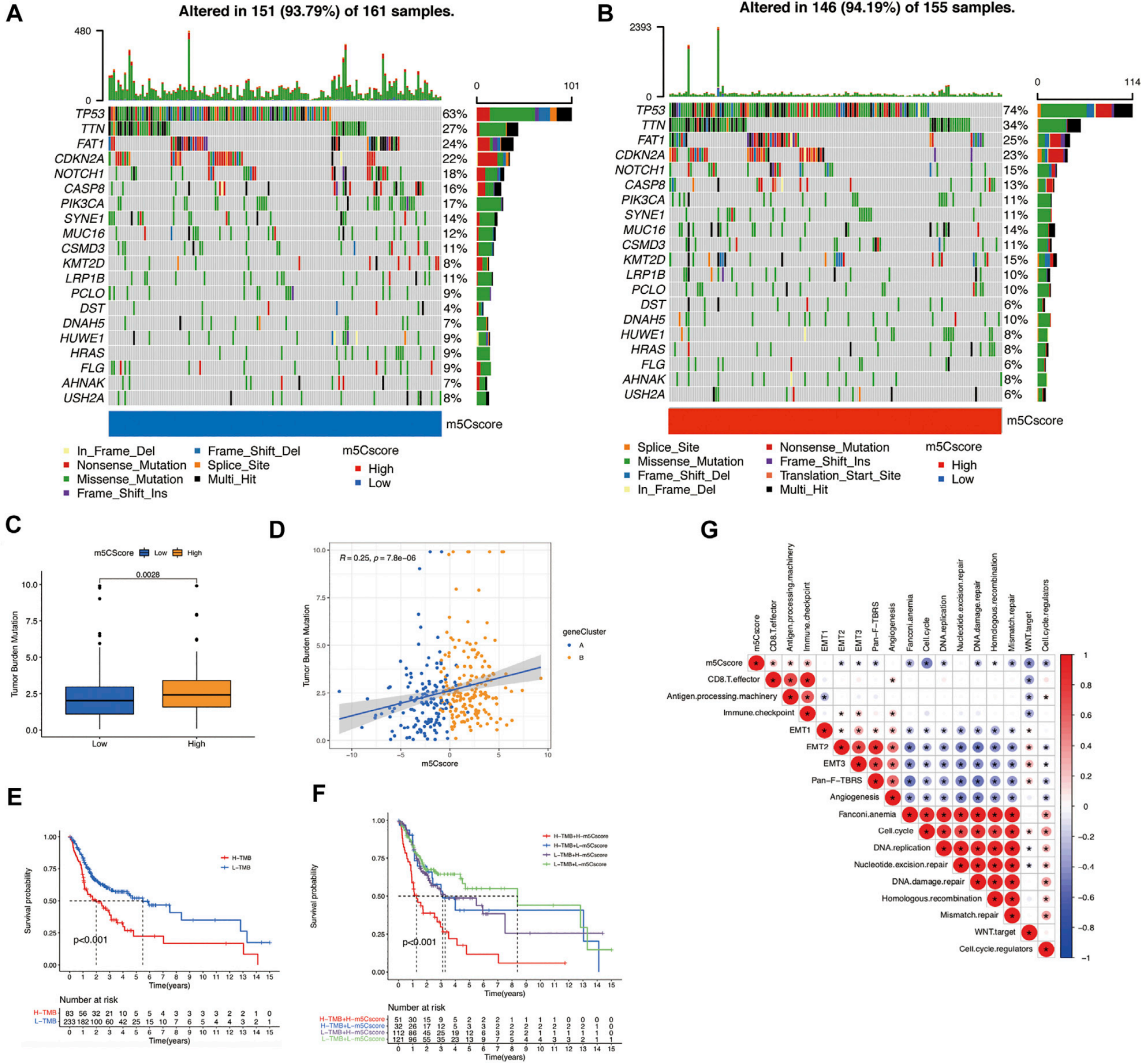
Exploration of m5Cscore and tumor mutation-related features. **(A)** and **(B)** Waterfall plots of the tumor mutation landscape in low **(A)** and high **(B)** m5Cscore groups of OSCC patients. **(C)** High-m5Cscore tumors were markedly correlated with a higher TMB (*p* < 0.0028, Student’s *t*-test). **(D)** There was a positive correlation between m5Cscore and TMB (*p* = 7.8e-06). **(E)** OS analysis for patients in the high- and low-TMB groups. **(F)** OS analysis for subgroup patients stratified by both m5Cscore and TMB quantification. **(G)** Correlations between m5Cscore and known biological gene signatures in OSCC patients. H, high; L, low; m5C, 5-methylcytosine; OS, overall survival; OSCC, oral squamous cell carcinoma. TMB, tumor mutational burden.

To verify the correlation between tumor mutational burden (TMB) and m5Cscores, we performed TMB quantification analysis in the high- and low-m5Cscore groups. In both m5Cscore groups, the somatic mutation rates of TP53, TTN, CDKN2A, NOTCH1, and CASP8 were relatively higher. Generally, the proportion and extent of somatic mutations in the high-m5Cscore group were higher and more extensive (Figures 5A,B). In addition, there was a high correlation between high TMB and high m5Cscore (*p* = 0.0028) (Figures 5C,D), as well as a worse prognosis (Figure 5E). The combination of a high m5Cscore and high TMB suggested an extremely poor OS for patients with OSCC (Figure 5F). Besides, m5Cscore was positively correlated with antigen processing machinery, CD8^+^ T effector, and immune checkpoint, but negatively correlated with cell cycle, Wnt target, mismatch repair, and cell cycle regulator by Spearman correlation analysis (Figure 5G).

### Predictive Effect of m5Cscore in OSCC Progression

**FIGURE 6:**
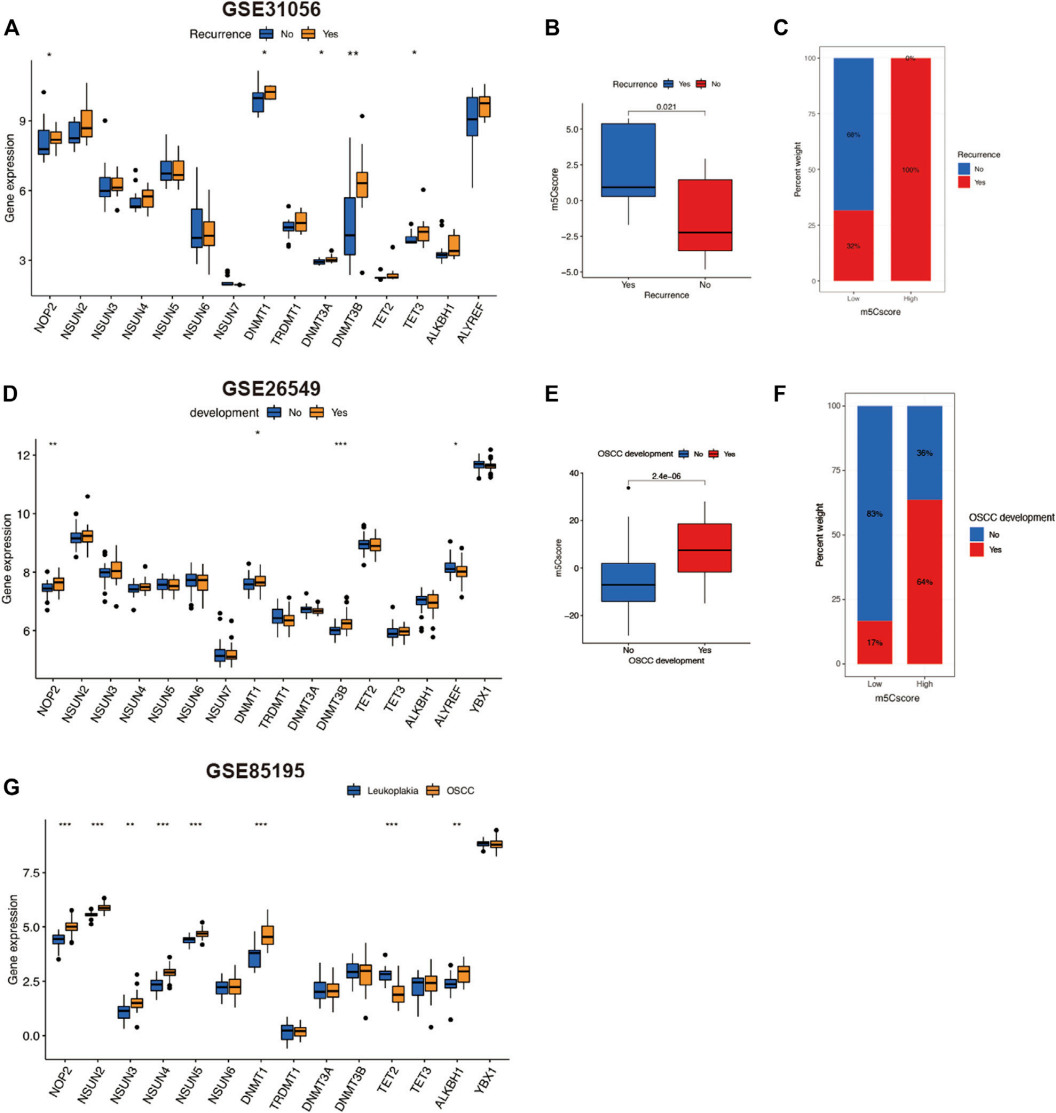
Predictive role of m5Cscore in OSCC development. **(A–C)** Analysis of recurrence in OSCC patients of GSE31056. **(A)** Comparison of gene expression of m5C regulators between patients with or without OSCC recurrent. **(B)** Comparison of m5Cscore between patients with or without OSCC recurrence. **(C)** Comparison of the proportion of OSCC recurrence between low- and high-m5Cscore groups. **(D–F)** Analysis of oral leukoplakia patients in GSE31056. **(D)** Comparison of gene expression of m5C regulators between patients with or without OSCC development. **(E)** Comparison of m5Cscore between patients with or without OSCC development. **(F)** Comparison of the proportion of OSCC development between oral leukoplakia patients with low and high m5Cscore. **(G)** Comparison of gene expression of m5C regulators between OSCC patients and oral leukoplakia patients in GSE85195. m5C, 5-methylcytosine; OSCC, oral squamous cell carcinoma.

We further investigated other predictive roles of m5Cscore in OSCC progression. Tumor recurrence is a crucial prognostic factor for OSCC. In this study, we compared the m5C regulators expression and m5Cscores between OSCC patients with and without recurrence in GSE31056. As illustrated in Figure 6A, there were five regulators—NOP2, DNMT1, DNMT3A, DNMT3B, and TET3—that were upregulated in OSCC patients with recurrence. Surprisingly, for patients with recurrence, m5Cscore was markedly higher than those without recurrence (Figure 6B). Moreover, 100% of patients in the high-m5Cscore group experienced recurrence in the course of OSCC (Figure 6C). We then analyzed the m5Cscore in patients with oral leukoplakia (Figures 6D–F). The m5Cscore was also markedly higher in patients with oral leukoplakia who developed OSCC. Three m5C regulators—NOP2, DNMT3B, and ALYREF—were upregulated (Figure 6D). We also verified the expression levels of m5C regulators in the microarray dataset GSE85195, which contained 34 OSCC patients and 15 oral leukoplakia patients. The expression of eight m5C regulators was upregulated in OSCC patients compared with that in oral leukoplakia patients (Figure 6G). These findings suggest that m5C methylation regulators might affect OSCC progression and that m5Cscore might has a predictive effect on the recurrence of OSCC and the deterioration of oral leukoplakia.

## Discussion

M5C-associated disorders are widely involved in the pathological process of many diseases, including cancer (Chen et al., 2019b). However, the effects of m5C on the occurrence and progression of OSCC are still unclear. In this study, we analyzed the roles of m5C regulators in the TIME and prognosis in OSCC and developed the m5Cscore scoring system. The results of these analyzes indicated m5C modification patterns and m5Cscore have potential predictive ability in the prognosis and progress of OSCC.

In this work, based on sixteen m5C regulators, two m5C modification patterns were identified. Compared to cluster A, cluster B was characterized by worse survival outcomes and higher expression levels of m5C regulators. Accumulated evidence reveals that OSCC is an immunosuppressive disease. In the TIME of OSCC, immune cells such as TILs, DCs, NK cells, and T-cells are in an inactive state (Jewett et al., 2006). Our ssGSEA analysis results showed that compared with cluster A, cluster B had lower immune activity in the above immune cell types, which suggests a more suppressive state of the TIME in cluster B. Recent reports have demonstrated that TILs widely affect the TIME in OSCC (Boxberg et al., 2019) (Quan et al., 2016) (Chen J. et al., 2021). Among the TILs, high expression of CD8^+^ T cells has been proven to be related to a better prognosis in HNSCC (Nakano et al., 2001). Moreover, a high CD4/CD8 ratio could be regarded as a marker of poor survival outcomes in cancer patients (Sato et al., 2005) (Quan et al., 2016). Our CIBERSORT and ssGSEA analysis results showed that the expression level of CD8^+^ T cells in cluster B were noticeably lower, while the expression level of CD4^+^ T cells was markedly higher than that in cluster A. Combined with the OS analysis results, our analysis results were in accordance with previous findings. Therefore, we speculated that m5C-related modification patterns might influence the TIME of OSCC, potentially affecting eventual survival outcomes.

We then constructed the m5Cscore scoring system to evaluate the m5C modification pattern in individual OSCC patients. Patients with a high m5Cscore had a more suppressive TIME state and worse OS outcomes compared with patients with a low m5Cscore. In addition, a higher m5Cscore was associated with poor survival outcomes and high TMB. These results indicate that m5Cscore may provide a new approach for predicting the TIME state and prognosis in OSCC.

For OSCC patients, locoregional recurrence after surgical excision is a problem that could negatively affect prognosis (Anderson et al., 2015). Recent studies have reported that the recurrence rate of OSCC was 16.0–32.7%; the survival rate was markedly lower in patients with local recurrence (33.3%), compared with that in patients without local recurrence (94.3%) (Yanamoto et al., 2012) (Wang et al., 2013). In this study, we analyzed the m5Cscore in OSCC patients with or without recurrence. Surprisingly, the m5Cscore in patients with recurrence was markedly higher, compared with those without recurrence. Five m5C regulators—NOP2, DNMT1, DNMT3A, DNMT3B, and TET3—were significantly upregulated in OSCC patients with recurrence. We speculate that there is a correlation between m5C methylation modification and OSCC recurrence, which is worthy of further in-depth study.

Approximately 0–36% of oral leukoplakia cases are expected to progress to OSCC (van der Waal, 2014) (Lee et al., 2000) (Chaturvedi et al., 2020). However, in clinics, there is still a lack of sensitive and effective methods to predict the deterioration of oral leukoplakia. Our analysis indicated that the m5Cscore was markedly higher in patients with oral leukoplakia who developed OSCC. The expression of three m5C regulators—NOP2, DNMT3B, and ALYREF—was upregulated in oral leukoplakia patients from two microarray datasets, GSE26549 and GSE85195. These three gene signatures may serve as deterioration biomarkers for patients with oral leukoplakia. However, further experimental and clinical experiments are needed to verify this hypothesis.

Human papillomavirus (HPV) infection was once thought to be a trigger for OSCC. However, recent studies have dismissed this viewpoint. No significant difference exists in the survival outcomes between HPV-positive and HPV-negative OSCC patients (Kansy et al., 2014) (Jiang and Dong, 2017). In this study, GSE65858 contained 70 HPV-negative and 13 HPV-positive OSCC patients. Our analysis results indicated that no significant difference existed in the prognosis (*p* = 0.109) and m5Cscore (*p* = 0.9) between HPV-negative and HPV-positive OSCC patients (Supplementary Figures S4G–H). In addition, the validation cohort GSE41613 containing 97 HPV-negative OSCC patients. We obtained almost consistent results for m5C-regulator expression, TIME characteristics, and survival outcomes in GSE41613, which suggested our findings is stable and reliable.

To summarize, this study revealed that m5C modification patterns play a crucial role in the regulation of the TIME in OSCC and can affect the prognosis and progress of OSCC patients. The constructed m5Cscore scoring system could predict the TIME state and the prognosis of individual OSCC patients, including the survival outcome, tumor recurrence, and deterioration of oral leukoplakia to OSCC. The m5C modification patterns and the m5Cscore scoring system might help in the prediction of the progress and prognosis of OSCC.

## Data Availability

The datasets presented in this study can be found in online repositories. The names of the repository/repositories and accession number(s) can be found in the article/Supplementary Material.
