# Supplementary material for: The RNA Methylation Modification 5-Methylcytosine Impacts Immunity Characteristics, Prognosis and Progression of Oral Squamous Cell Carcinoma by Bioinformatics Analysis
Source: Front Bioeng Biotechnol. 2021 Dec 9;9:760724. doi: 10.3389/fbioe.2021.760724 (PMC8696036; doi:10.3389/fbioe.2021.760724)
Supplement: Supplementary file 2 [file Table1.DOCX]

**The RNA methylation modification 5-methylcytosine impacts immunity characteristics and prognosis in oral squamous cell carcinoma**

**Supplementary Information**

**Figure S1** Survival analysis of m5C regulators in OSCC patients in the validation cohort. m5C, 5-methylcytosine; OSCC, oral squamous cell carcinoma.

**Figure S2** Unsupervised consensus clustering of m5C regulators in OSCC patients. **(A)**-**(C)** Unsupervised consensus clustering of OSCC patients. Consensus matrices of the training cohort for k = 2 (A), consensus clustering cumulative distribution function (CDF) (B), and relative change in area under the CDF curve (C). **(D)**-**(F)** Unsupervised consensus clustering of OSCC patients in the validation cohort. Consensus matrices of the training cohort for k = 2 (D), consensus clustering CDF (E), and relative change in area under the CDF curve (F). **(G)** Comparison of m5C expression between two m5C clusters. *P < 0.05, ***P < 0.001. m5C, 5-methylcytosine; OSCC, oral squamous cell carcinoma.

**Figure S3** Unsupervised clustering of m5C phenotype-related genes in OSCC patients. **(A)** Selection of the soft threshold made the index of scale-free topologies reach 0.80. **(B)-(D)** Unsupervised consensus clustering of m5C phenotype-related genes. Consensus matrices of the training cohort for k = 2 (B), consensus clustering CDF (C), the relative change in area under the CDF curve (D). **(E)**-**(G)** Unsupervised consensus clustering of m5C phenotype-related genes in validation cohort. Consensus matrices of the training cohort for k = 2 (E), consensus clustering cumulative distribution function (CDF) (F), the relative change in area under the CDF curve (G). **(G)** Comparison of m5C expression between two m5C gene clusters. *P < 0.05, **P < 0.01; ***P < 0.001. CDF, cumulative distribution function; m5C, 5-methylcytosine; OSCC, oral squamous cell carcinoma.

**Figure S4** Exploration of the relevance of clinical features of m5Cscore in OSCC patients. **(A)**-**(B)** Survival outcomes of low and high m5Cscore in OSCC patients at T1–T2 (A) and T3–T4 stages (B). (**C)**-**(D)** Survival outcomes of low and high m5Cscore in OSCC at N0 (C) and N1–N3 stage (D). **(E)** Differences in m5Cscore between the two m5C modification patterns in OSCC patients (P < 2.22e−16). **(F)** Differences in m5Cscore between the two gene clusters in OSCC patients. (P < 2.22e−16). **(G)** OS analysis of HPV-positive and HPV-negative OSCC patients in GSE65858 (P = 0.109, log-rank test). **(H)** Comparison of m5Cscore in HPV-positive and HPV-negative OSCC patients in GSE65858 (P = 0.9). HPV, human papillomavirus; m5C, 5-methylcytosine; OS, overall survival; OSCC, oral squamous cell carcinoma.

**
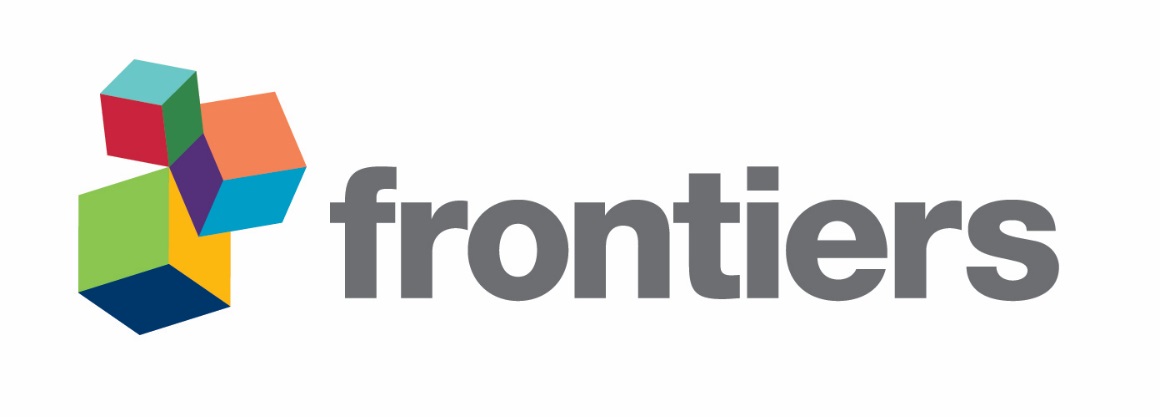
**
