# Supplementary figures and images for: The RNA Methylation Modification 5-Methylcytosine Impacts Immunity Characteristics, Prognosis and Progression of Oral Squamous Cell Carcinoma by Bioinformatics Analysis
Source: Front Bioeng Biotechnol. 2021 Dec 9;9:760724. doi: 10.3389/fbioe.2021.760724 (PMC8696036; doi:10.3389/fbioe.2021.760724)

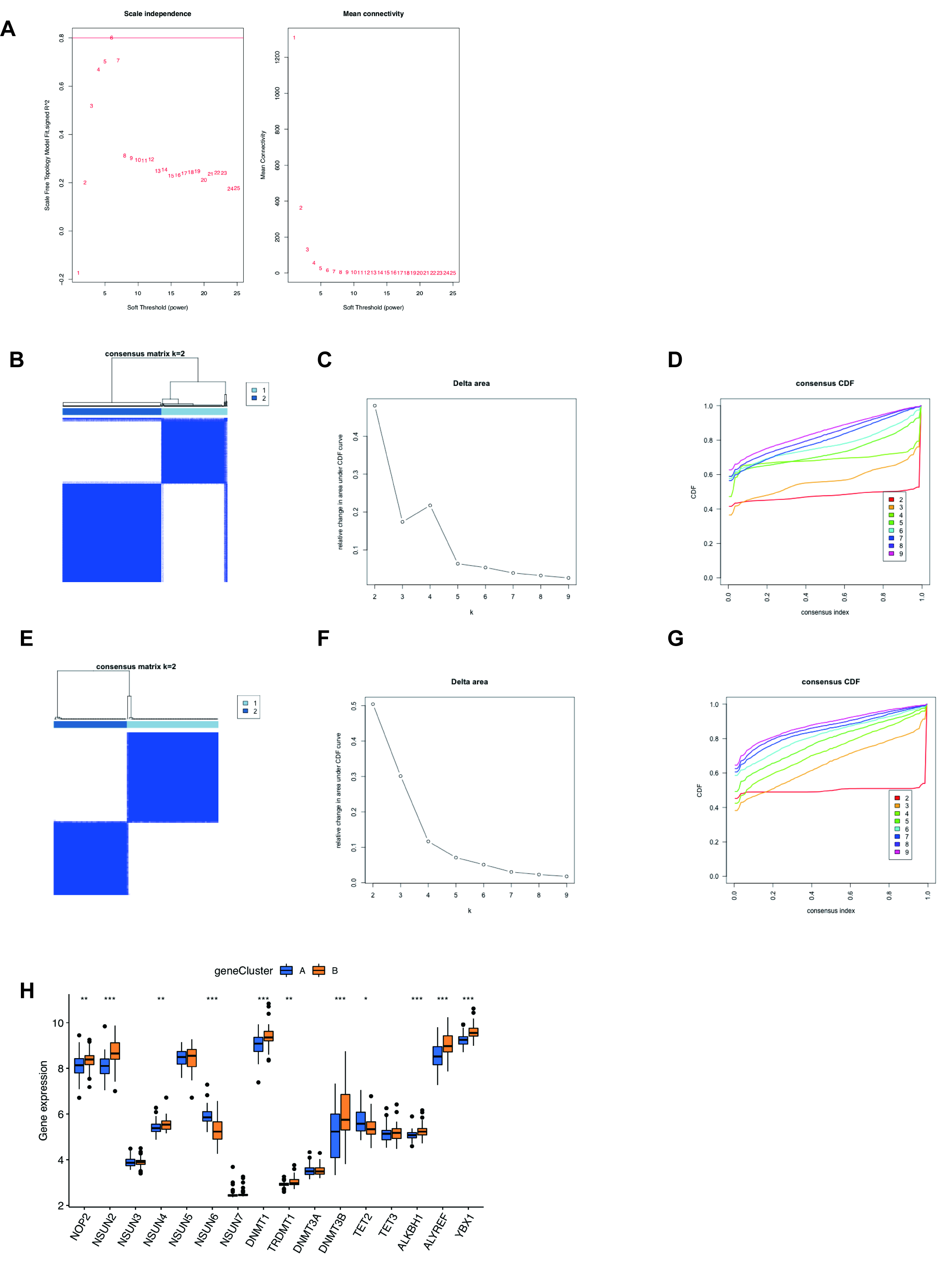

Supplement: Supplementary file 3 [file Image3.TIF]

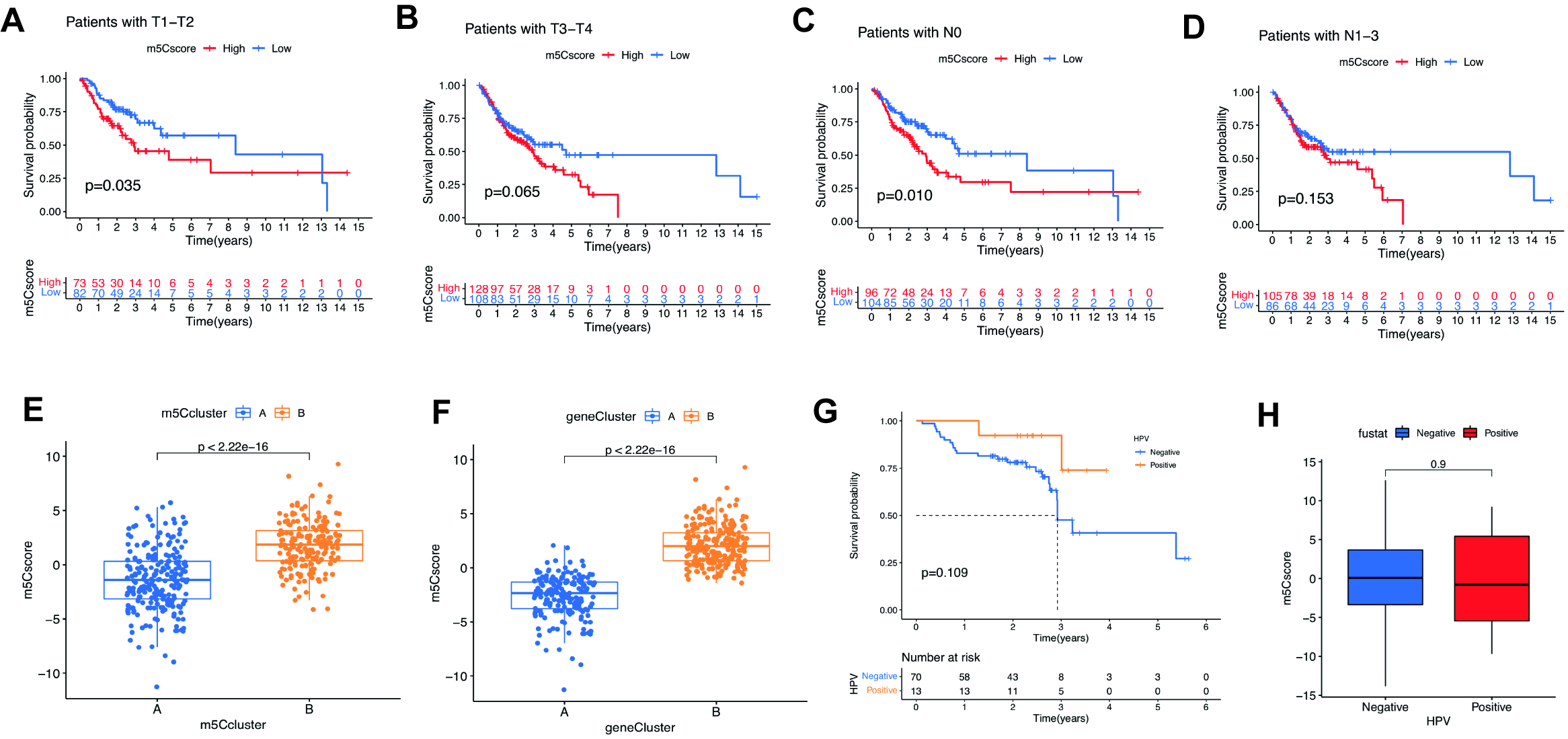

Supplement: Supplementary file 4 [file Image4.TIF]

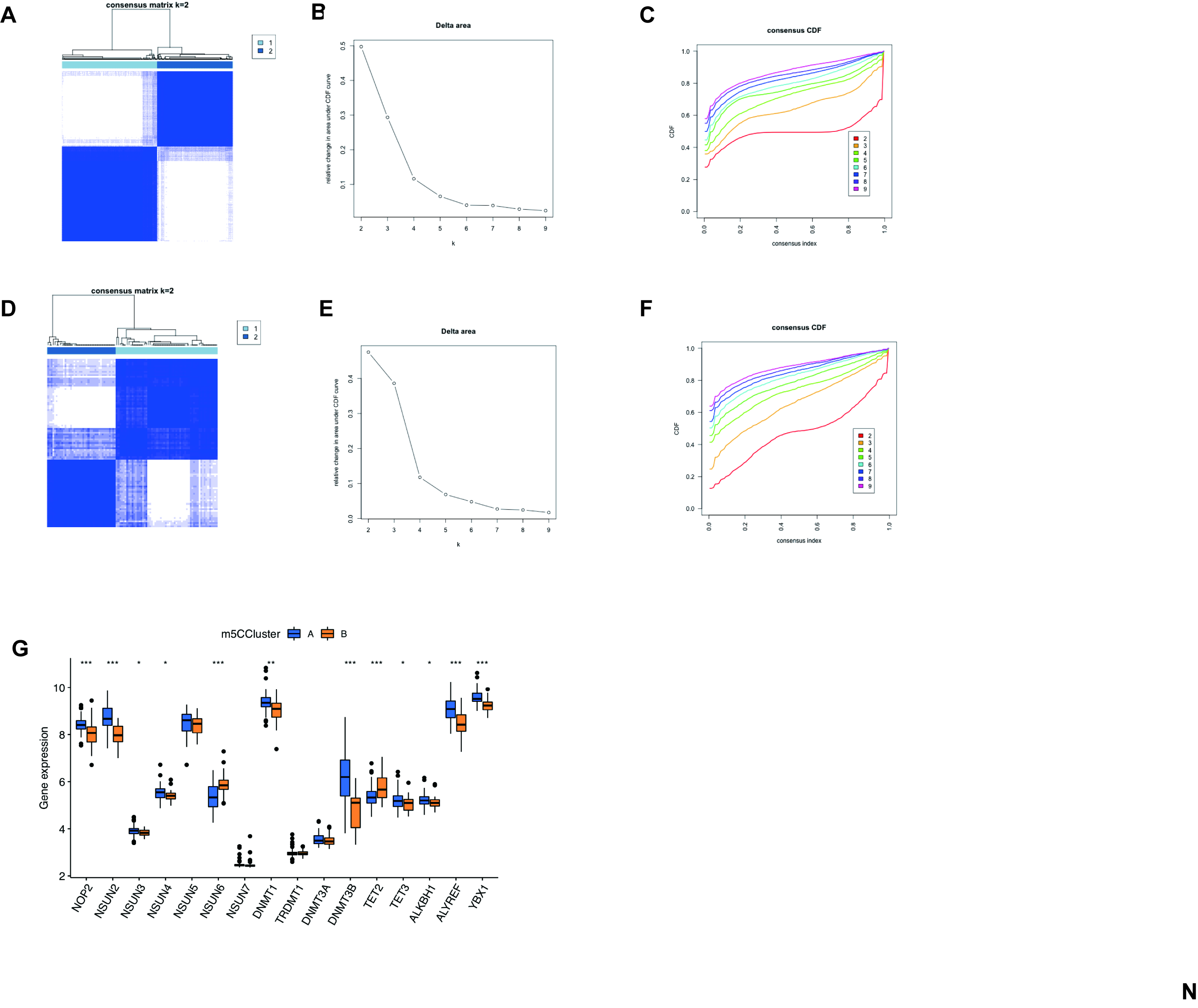

Supplement: Supplementary file 5 [file Image2.TIF]

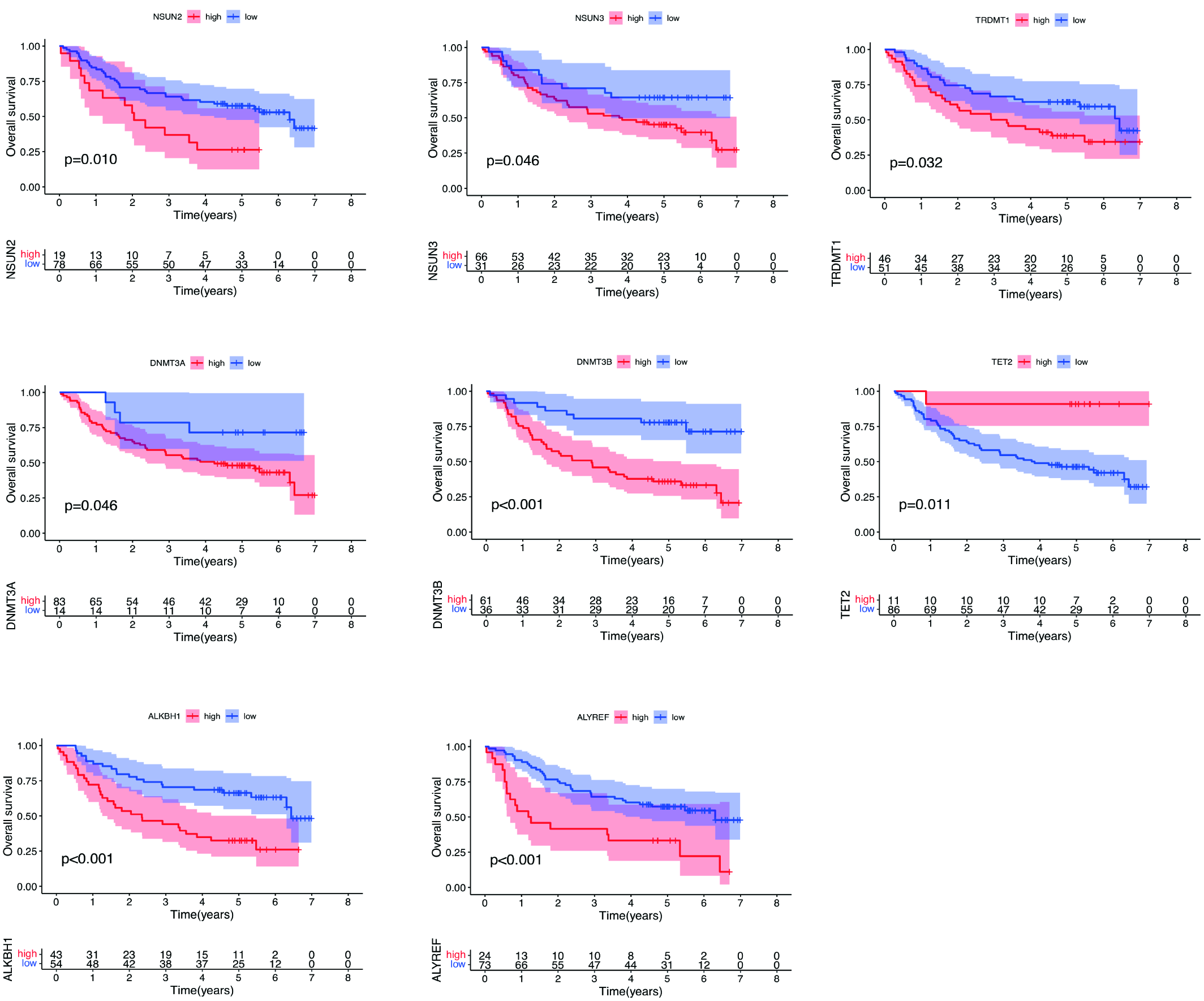

Supplement: Supplementary file 6 [file Image1.TIF]
